# S100A8, S100A9 and S100A8/A9 heterodimer as novel cachexigenic factors for pancreatic cancer-induced cachexia

**DOI:** 10.1186/s12885-023-11009-8

**Published:** 2023-06-06

**Authors:** Wei-Chih Liao, Chih-Ta Chen, You-Shu Tsai, Xin-Ya Wang, Yen-Tzu Chang, Ming-Shiang Wu, Lu-Ping Chow

**Affiliations:** 1 Division of Gastroenterology and Hepatology, Department of Internal Medicine, National Taiwan University Hospital, Taipei, Taiwan; 2 Internal Medicine, College of Medicine, National Taiwan University, Taipei, Taiwan; 3 Graduate Institute of Biochemistry and Molecular Biology, College of Medicine, National Taiwan University, No.1, Jen-Ai Road Section 1, Taipei, 10051 Taiwan

**Keywords:** Pancreatic cancer, Cachexia, Muscle atrophy, S100A8, S100A9, Biomarker

## Abstract

**Background:**

Cancer cachexia, occurring in ~ 80% pancreatic cancer (PC) patients overall, is a paraneoplastic syndrome mediated by cancer-induced systemic inflammation and characterized by weight loss and skeletal muscle wasting. Identifying clinically relevant PC-derived pro-inflammatory factors with cachexigenic potential may provide novel insights and therapeutic strategies.

**Methods:**

Pro-inflammatory factors with cachexigenic potential in PC were identified by bioinformatic analysis. The abilities of selected candidate factors in inducing skeletal muscle atrophy were investigated. Expression levels of candidate factors in tumors and sera was compared between PC patients with and without cachexia. Associations between serum levels of the candidates and weight loss were assessed in PC patients.

**Results:**

S100A8, S100A9, and S100A8/A9 were identified and shown to induce C2C12 myotube atrophy. Tumors of PC patients with cachexia had markedly elevated expression of S100A8 (P = 0.003) and S100A9 (P < 0.001). PC patients with cachexia had significantly higher serum levels of S100A8, S100A9 and S100A8/A9. Serum levels of these factors positively correlated with percentage of weight loss [correlation coefficient: S100A8: 0.33 (P < 0.001); S100A9: 0.30 (P < 0.001); S100A8/A9: 0.24 (P = 0.004)] and independently predicted the occurrence of cachexia [adjusted odds ratio (95% confidence interval) per 1ng/ml increase: S100A8 1.11 (1.02–1.21), P = 0.014; S100A9 1.10 (1.04–1.16), P = 0.001; per 1 µg/ml increase: S100A8/A9 1.04 (1.01–1.06), P = 0.009].

**Conclusions:**

Atrophic effects of S100A8, S100A9, and S100A8/A9 indicated them as potential pathogenic factors of PC-induced cachexia. In addition, the correlation with the degree of weight loss and prediction of cachexia in PC patients implicated their potential utility in the diagnosis of PC-induced cachexia.

**Supplementary Information:**

The online version contains supplementary material available at 10.1186/s12885-023-11009-8.

## Background

Pancreatic cancer (PC) is the 7th leading cancer deaths worldwide and projected to become the second leading cancer deaths in US by 2030 [1, 2]. Besides a high risk of recurrence after surgical resection and limited response to systemic therapies, an important contributor to the poor survival of PC is PC-induced cachexia [3]. Whereas cachexia secondary to other types of cancer mostly occurs at an advanced stage, the prevalence of cachexia at the diagnosis of PC reaches approximately 60% and is not influenced by cancer stage [3]. Overall, cachexia develops in approximately 80% of PC patients during the disease course and adversely impacts treatment response and survival [3], with one-third of PC patients dying from cachexia-associated complications including impaired immunity and cardiopulmonary dysfunction [4, 5]. Regrettably, the critical determinants of PC-induced cachexia remain obscure and no effective treatment exists, underscoring the pressing need to find significant drivers and hence helping discover potential treatment strategies.

Cancer cachexia is a paraneoplastic syndrome mediated by cancer-induced systemic inflammation and characterized by refractory weight loss and skeletal muscle wasting [6, 7]. Notably, skeletal muscle atrophy results in increased circulating amino acids to fuel tumor growth and predicts poor survival independent of weight loss [8, 9]. Various pro-inflammatory factors such as TNF-α and IL-6 have been implicated in cancer cachexia, but the circulating levels of those factors do not differ significantly between PC patients with and without cachexia [10–13]. Collectively, the key pro-inflammatory factors that mediate systemic inflammation and thereby drive PC-induced cachexia remain elusive [14]. Therefore, identifying novel PC-derived cachexigenic factors may provide insights into the pathogenesis of PC-induced cachexia and yield novel therapeutic strategies.

In this study, we utilized bioinformatic approach to discover PC-derived pro-inflammatory factors with cachexigenic potential. The abilities of the selected candidate secreted proteins in inducing skeletal muscle atrophy were investigated. To ascertain the clinical relevance of those factors, expression of the potential cachexigenic factors was compared between patients with and without cachexia at the time of PC diagnosis, and the associations between those factors and the risk of cachexia and cachexia-related parameters were further analyzed in PC patients.

## Methods

### Study design

**Fig. 1:**
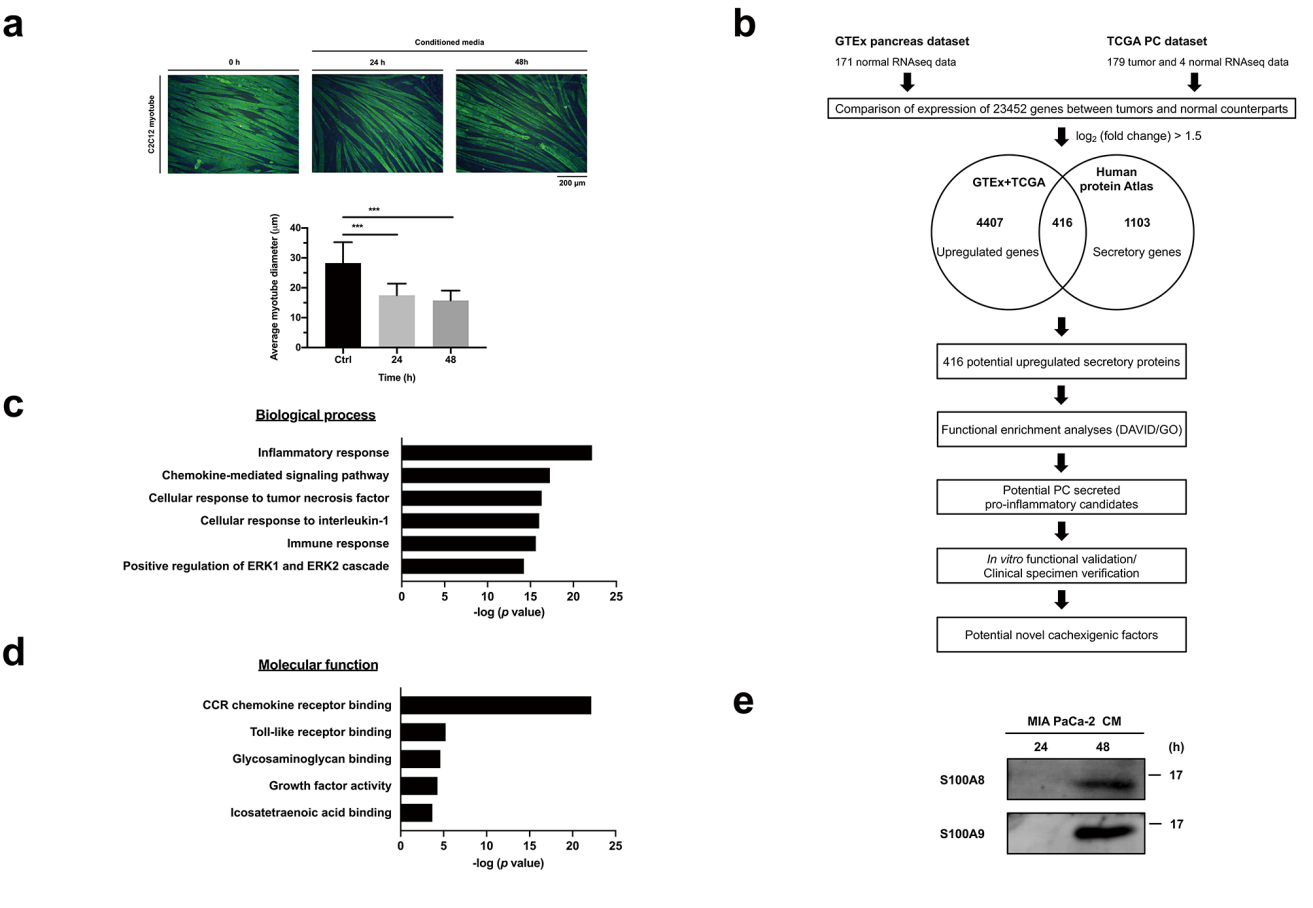
Identification of potential cachexigenic factors in pancreatic cancer (PC)-induced cachexia. **(a)** Atrophic effects of conditioned media (CM) from MIA PaCa-2 PC cells on C2C12 myotubes. CM was harvested by incubation of MIA PaCa-2 cells in serum-free DMEM for 48 h. Differentiated C2C12 myotubes were treated by 33% CM for the indicated time. Morphological changes were examined by immunofluorescence staining and quantified. Microscopic images (200x) were captured with a fluorescence microscope and diameters of the myotubes were measured and quantified. Green, anti-myosin heavy chain; blue, cell nuclei. Data are presented as means ± SD. ***P < 0.001. **(b)** Workflow for the identification of potential PC-derived cachexigenic factors via bioinformatic big data mining. GTEx, Genotype-Tissue Expression; TCGA, The Cancer Genome Atlas. **(c)** Biological process enrichment of potential PC-derived secreted proteins via Database, Annotation, Visualization and Integrated Discovery. **(d)** Molecular function annotation of inflammatory response-associated proteins via Gene Ontology. **(e)** Validation of the secretion of S100A8 and S100A9 by PC cells. CM harvested at 24 and 48 h were analyzed via immunoblotting for examination of S100A8 and S100A9 secretion

We first validated the potential of PC-derived secretome from MIA PaCa-2 cells (ATCC, Manassas, VA, USA) in inducing muscle atrophy. The subsequent strategy for identifying potential PC-derived cachexigenic factors is summarized in Fig. 1b. Bioinformatic analyses for discovering potential cachexigenic factors were performed by comparing gene expression between normal pancreas from the Genotype-Tissue Expression (GTEx) database and PC from The Cancer Genome Atlas (TCGA) database via GEPIA (http://gepia.cancer-pku.cn) [15]. 4823 upregulated genes (${\text{L}\text{o}\text{g}}_{2} \left(\text{F}\text{o}\text{l}\text{d} \text{c}\text{h}\text{a}\text{n}\text{g}\text{e}\right)>1.5$) in the PC tissue were further analyzed to extract secreted candidates by comparing with the dataset in Human Protein Atlas (https://www.proteinatlas.org/humanproteome/tissue/secretome). Subsequently, 416 potential secreted candidates were analyzed with Database for Annotation, Visualization and Integrated Discovery (https://david.ncifcrf.gov/home.jsp) and Gene Ontology (http://geneontology.org) for functional enrichment and molecular annotation. Expression of potential cachexigenic factors in PC tissues was assessed by immunohistochemistry, and their serum levels were measured by ELISA and compared among groups of healthy control, pancreatitis and PC patients with and without cachexia.

### MIA PaCa-2 conditioned medium (CM)

CM from MIA PaCa-2 cells was collected and processed as previously described [16]. Briefly, equal number of MIA PaCa-2 cells were seeded onto 10 cm culture plates, incubated overnight, and washed with PBS and incubated in serum free medium for 24 or 48 h. Supernatants were collected, centrifuged and filtered with 0.22 μm syringe filter for removing cell debris and sterilization. The collected CM was subsequently used for morphological and immunoblotting analyses. In the examination of S100A8 and S100A9 secretion by PC cells in CM, equal volumes of CM incubated for 24 or 48 h were subjected to immunoblotting analysis.

### Immunoblotting

Commercial antibodies used for immunoblotting in this study were S100A8 (Santa Cruz Biotechnology, CA, USA); S100A9 (Genetex, Irvine, CA, USA); Atrogin-1 (Abcam, Cambridge, UK); MuRF1 (Abcam, Cambridge, UK) and GAPDH (Santa Cruz Biotechnology, CA, USA). Protein extracts were separated by SDS-PAGE and transferred onto polyvinylidene fluoride membrane. For detection of target proteins, the skimmed milk blocked membranes were incubated with specified primary antibodies and corresponding secondary antibodies conjugated with horseradish peroxidase. The bound antibodies were detected using enhanced chemiluminescence reagent (Merck Millipore, Burlington, MA, USA). Quantification was performed by densitometry using ImageQuant version 5.2 software (GE Healthcare, Chicago, IL, USA).

### Preparation of recombinant candidate proteins

Recombinant His-tagged S100A9 and galectin-1 were prepared as described previously [16]. Target genes were cloned into pET28a vector (Novagene, Bedford, MA, USA) and expressed in Escherichia coli strain BL21 after isopropyl b-D-1-thiogalactopyranoside induction and purified by a Ni^2+^-chelating Sepharose column (GE Healthcare, Chicago, IL, USA). The endotoxin was removed by dialysis against buffer with 1% Triton X-114 before use. S100A8 (ProSpec, Rehovot, Israel) and S100A8/A9 (BioLegend, San Diego, CA, USA) were purchased from commercial sources.

### C2C12 myotube morphological analysis

C2C12 myoblasts (ATCC, Manassas, VA, USA) were grown in DMEM (HyClone, Marlborough, MA, USA) supplemented with 10% FBS (ThermoFisher, Rockford, IL, USA) and 1% Penicillin/Streptomycin (ThermoFisher) into 24-well culture plates until reaching 90% confluence for 2 days. The cells were then differentiated in DMEM containing 2% horse serum for 3 days. After myotube formation, cells are treated with CM, S100A8, S100A9 or S100A8/A9 for the indicated doses and time. The morphological changes of C2C12 myotubes were examined via immunofluorescent staining using a specific anti-myosin heavy Chain antibody (R&D systems, Minneapolis, MN, USA), as previously described [17]. Briefly, C2C12 myotubes were fixed, blocked, and immuno-stained with an antibody against myosin heavy chain followed by a Fluorescein-isothiocyanate (FITC)-labeled secondary antibody. Nuclear staining was performed with 4,6-diamidino-2-phenylindole (DAPI), and images were captured using an Olympus IX71 fluorescence microscope (Olympus, Tokyo, Japan) at a magnification of 200x. At least 5 images were obtained for each experimental condition. The mean myotube diameter for each condition was determined by calculating the average width of all myotubes, which included both prominently extended structures and cell fusions with at least 5 nuclei, in all quantified images using Image J software (NIH, Bethesda, MD, USA).

### Patients

The study was approved by the National Taiwan University Hospital Research Ethics Committee (201909057RINA) and was conducted in accordance with the Declaration of Helsinki. Patients included in this study (n = 143) were diagnosed with pancreatic adenocarcinoma by histology/cytology at a tertiary referral center (National Taiwan University Hospital) and consecutively enrolled between September 2006 and January 2019 with required blood sample and clinical information including cachexia-related parameters needed for this study. Tumor stage was defined according to the eighth edition tumor, node, metastasis (TNM) system of the combined American Joint Committee on Cancer (AJCC)/Union for International Cancer Control (UICC) [18]. Patients with acute or chronic pancreatitis (n = 50) and healthy controls (n = 50) were also included for comparison. Acute pancreatitis was diagnosed by fulfilling two or more of the following criteria: (1) consistent acute abdominal pain; (2) elevated serum lipase/amylase level greater than 3 times the upper limit of normal; (3) characteristic findings on abdominal imaging studies [19]. Chronic pancreatitis was diagnosed based on parenchymal and/or intraductal calcifications and dilatation of the pancreatic duct on imaging studies or histology [20]. Healthy controls were selected from subjects who were considered to be free of any malignancy and acute or chronic pancreatitis after a comprehensive health examination at the same center, including evaluation of symptoms and medical history, physical examination, blood tests, radiological and endoscopic examinations, and abdominal sonography [21].

### Ascertainment of weight loss, skeletal muscle mass, and cachexia

Cachexia was defined as weight loss > 5% within 6 months, or weight loss > 2% in individuals with body mass index < 20 kg/m^2^, or the coexistence of weight loss > 2% and sarcopenia. Lumbar skeletal muscle index (SMI), which is linearly correlated with whole-body muscle mass and the preferred method for muscle mass assessment, was calculated from computed tomography (CT) images obtained at the diagnosis of PC as previously described, with values < 39.8 cm^2^/m^2^ in men and < 28.4 cm^2^/m^2^ in women considered as sarcopenia in Asians [22]. Briefly, two consecutive CT images containing the third lumbar vertebrae (L3) were used for measurement. Skeletal muscles at the L3 level including psoas, paraspinal muscles (erector spinae and quadratus lumborum), and abdominal wall muscles (transversus abdominis, external and internal obliques, and rectus abdominis) were identified using Hounsfield unit thresholds of -29 to + 150. The sum of cross-sectional areas of these muscles on each image was computed, and the mean value of the two images was taken as total area of L3 skeletal muscles and further normalized for stature to yield the L3 SMI. Information on the degree of weight loss within 6 months before the diagnosis of PC was obtained by patient recall at enrollment, and medical records, where available, were reviewed to reduce inaccuracies in patient recall. The percentage of weight loss within 6 months was calculated as weight lost within 6 months divided by the sum of weight loss within 6 months and weight at diagnosis. If weight loss within 6 months was less than 0.5 kg according to patient recall and/or medical records, body weight lost was recorded as 0.

### Measurement of serum levels of S100A8, S100A9 and S100A8/A9 heterodimer

Blood samples were collected after an overnight fast, and those of PC patients were collected at cancer diagnosis before initiation of treatment for PC. Serum was separated by centrifugation and stored at -80 °C until use, and all samples were coded for blind analysis. Serum levels of S100A8, S100A9 and S100A8/A9 heterodimer were determined by sandwich ELISA (R&D Systems, Minneapolis, MN, USA) as previously described [16].

### Immunohistochemistry

Tumor tissue samples were processed for paraffin embedding and 5-mm sections were prepared. Standard avidin-biotin immunohistochemical analyses of the sections were performed according to the manufacturer’s recommendations (Leica Biosystems, Newcastle, U.K.). In brief, tissue sections were deparaffinized, rehydrated, heated for antigen retrieval, blocked, and reacted with primary antibodies, including S100A8 (Santa Cruz Biotechnology), S100A9 (Genetex) and S100A8/A9 (R&D Systems), secondary antibodies, and substrate 3,3’-diaminobenzidine. In addition, the sections were counterstained with hematoxylin. Expression of S100A8, S100A9 and S100A8/A9 heterodimer was scored using an H score ranging from 0 to 3, defined as [(% of 0) x 0] + [(% of 1+) x 1] + [(% of 2+) x 2] + [(% of 3+) x 3] (0, nil; 1+, low or weak; 2+, moderate; 3+, high or strong; %, percent of tumor cells of a certain intensity).

### Statistical analysis

Normally distributed variables were summarized as $\text{m}\text{e}\text{a}\text{n}\pm \text{S}\text{D}$, whereas variables that were not normally distributed were summarized as median (interquartile range). Mann–Whitney U-test was used to compare continuous variables between groups, and Dunn’s test was used for multiple comparisons. Categorical variables were compared with Fisher exact test. Spearman correlation coefficient was used to assess correlations between serum levels and H scores or percentage of body weight loss. Associations between the risk of cachexia and serum levels of candidate cachexigenic factors and clinical characteristics were analyzed using logistic regression, and variables with P values < 0.2 in univariable analyses were included in the multivariable analysis. All tests were two-tailed and P values less than 0.05 were considered as statistically significant. Statistical analyses were performed using Stata17 (StataCorp, College Station, TX, USA) and GraphPad Prism version 9.3.1 (GraphPad Software, San Diego, CA, USA).

## Results

### Identification of candidate mediators of muscle wasting in PC-induced cachexia

We first assessed whether the CM of MIA PaCa-2 cells could induce atrophy of skeletal muscle cells. As shown in Fig. 1a, CM of MIA PaCa-2 cells significantly reduced the diameter of C2C12 myotubes compared with control, supporting the concept that PC cells secrete cachexigenic factors that could induce muscle atrophy. Bioinformatic analysis of differential gene expression between normal pancreas and PC yielded 4,823 upregulated genes, and 416 of those encode proteins potentially secreted by PC tissue according to Human Protein Atlas (Fig. 1b). Biological process enrichment analysis of the 416 upregulated PC-derived molecules by Database for Annotation, Visualization and Integrated Discovery showed that inflammatory response was the top-ranked biological process (n = 50; Fig. 1c and Table S1), and further molecular function annotation and ranking of those 50 molecules via Gene Ontology identified CCR chemokine receptor binding (n = 13) and Toll-like receptor binding (n = 3) as the two major inflammation-related categories (Fig. 1d; Table 1). Based on the notion that cachexigenic factors might be linked to chronic inflammation, we selected S100A8 and S100A9 as candidates for further investigation and verified their existence in the CM of MIA PaCa-2 cells (Fig. 1e).

**Table 1:** Molecular function annotation of the top-ranked inflammatory response-related molecules (n = 50) via Gene Ontology

Molecular function	No. of genes	P value	Genes
CCR chemokine receptor binding	13	8.99E-23	CCL3, CCL13, CCL19, CCL4, CCL20, CCL21, CCL26, CCL24, CCL18, CXCL13, CCL15, CCL17, CCL22
Toll-like receptor binding	3	6.33E-06	S100A9, LY96, S100A8
Glycosaminoglycan binding	6	2.7E-05	CXCL10, TNFAIP6, CXCL6, CXCL13, THBS1, CCL15
Growth factor activity	5	5.67E-05	BMP2, MIF, TGFB1, IL34, CXCL1
Icosatetraenoic acid binding	2	2.13E-04	S100A9, S100A8

### Atrophic effects of candidate cachexigenic factors on skeletal muscle cells

**Fig. 2:**
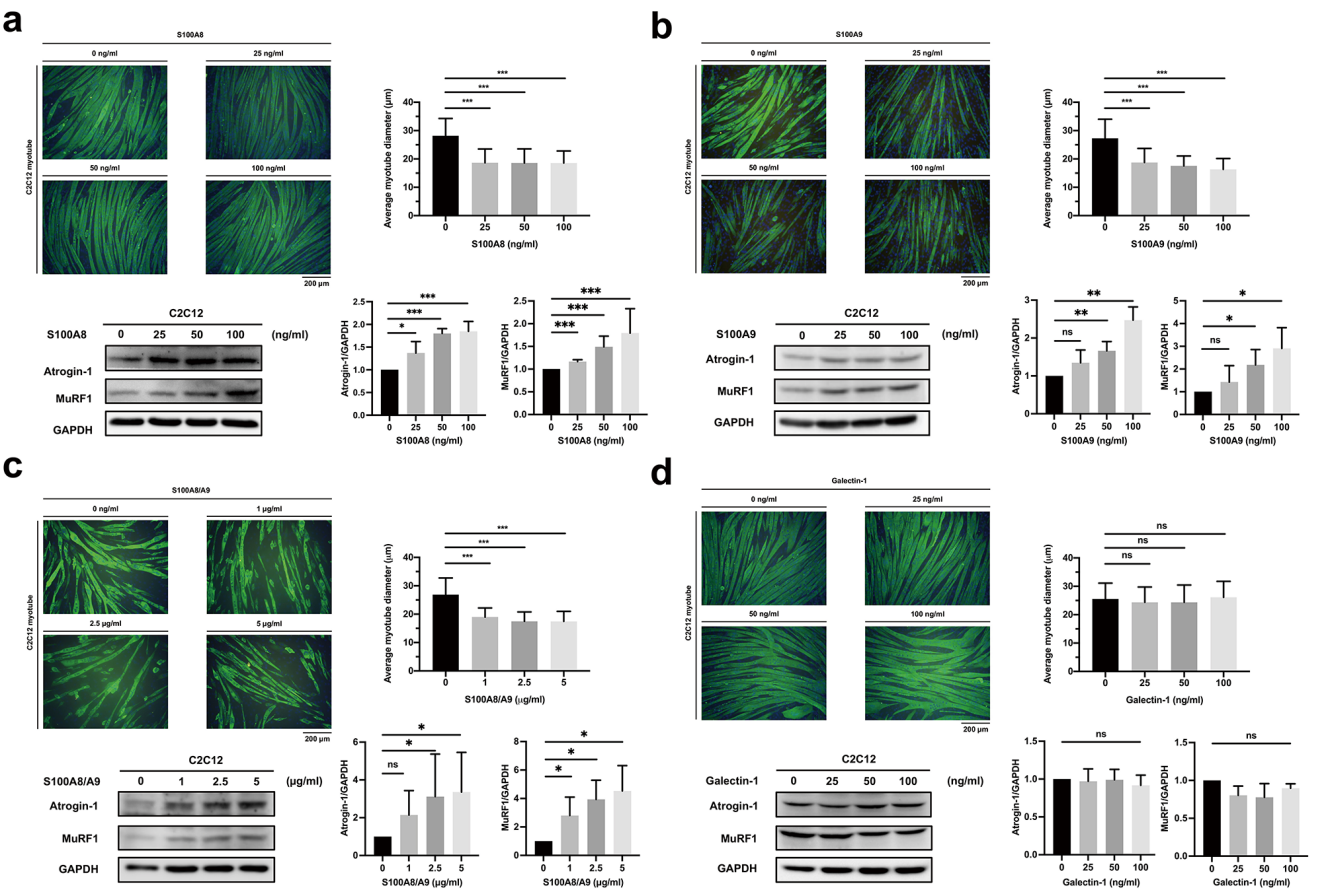
Atrophic effects of potential cachexigenic factors on C2C12 myotubes. Differentiated C2C12 myotubes were treated with **(a)** S100A8, **(b)** S100A9, **(c)** S100A8/A9 heterodimer, and **(d)** galectin-1 as a negative control at indicated concentrations for 24 h. Morphological changes and expression of muscle-specific protein degradation markers Atrogin-1 and MuRF1 were examined by immunofluorescence staining and immunoblotting, respectively. Microscopic images (200x) were captured with a fluorescence microscope and diameters of the myotubes were measured and quantified. Green, anti-myosin heavy chain; blue, cell nuclei. Data are presented as means ± SD. ns, not significant; *P < 0.05; **P < 0.01; ***P < 0.001

To determine whether the selected candidate proteins can induce cachexia, we assessed their effects on skeletal muscle cells in vitro. Treatment with S100A8 and S100A9 significantly reduced the diameter of C2C12 myotubes in all indicated concentrations (Fig. 2a, b). As S100A8 and S100A9 form stable heterodimers under physiological conditions [23], the effects of S100A8/A9 heterodimer on C2C12 cells were examined and similar results were observed (Fig. 2c). In contrast, galectin-1, previously identified as a biomarker of PC [24, 25], did not induce myotube atrophy and was used as a negative control (Fig. 2d). Collectively, S100A8, S100A9 and S100A8/A9 heterodimer induced atrophy of skeletal muscle cells and thus were potential cachexigenic factors.

### S100A8, S100A9 and S100A8/A9 heterodimer promotes muscle atrophy via upregulation of Atrogin-1 and MuRF1 in C2C12 myotubes

Increasing expression of muscle-specific E3 ubiquitin ligases Atrogin-1 and MuRF1 is a hallmark mechanism promoting muscle atrophy [7]. We investigated whether the selected candidates could mediate muscle atrophy via upregulation of these two muscle-specific protein degradation enzymes. Treating C2C12 myotubes with the S100A8, S100A9 and S100A8/A9 significantly increased the expression of Atrogin-1, and MuRF1 in a dose-dependent manner, whereas treatment with galectin-1 did not (Fig. 2a-d). These results suggest that S100A8, S100A9 and S100A8/A9 might induce muscle cell atrophy via upregulation of Atrogin-1 and MuRF1.

### Expression of S100A8, S100A9, and S100A8/A9 heterodimer in tumors with PC-induced cachexia

**Fig. 3:**
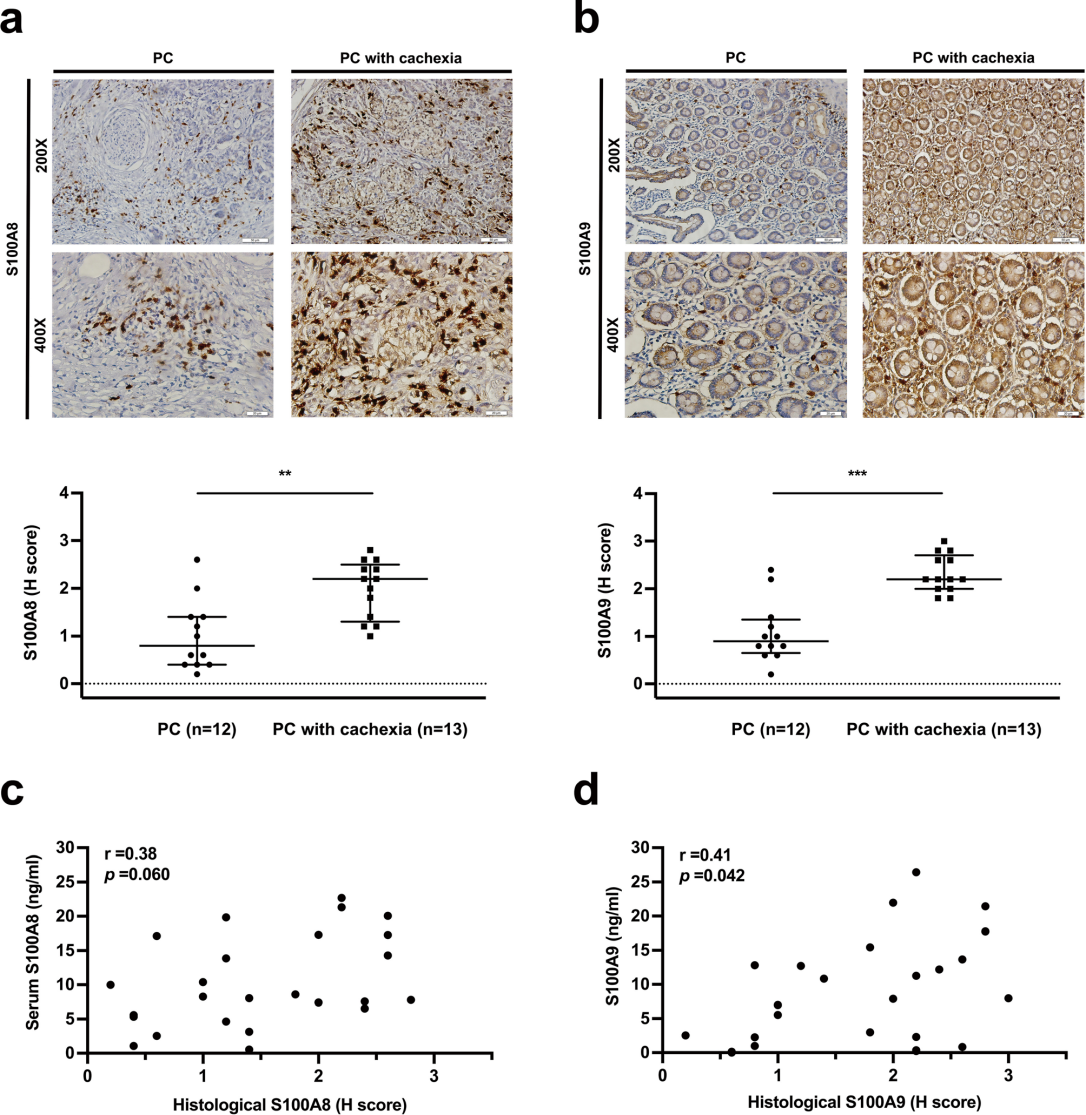
Expression and correlations with serum levels of potential cachexigenic factors in tumors of pancreatic cancer (PC) patients with or without cachexia. **(a, b)** The expression levels of S100A8 **(a)** and S100A9 **(b)** was examined by immunohistochemistry and quantified. Representative images were presented. **(c, d)** The correlations between histological and serum levels of S100A8 **(c)** and S100A9 **(d)** were evaluated by Spearman’s correlation coefficient (r). **P < 0.01; ***P < 0.001

We next examined the expression of S100A8, S100A9 and S100A8/A9 heterodimer in paraffin-embedded resected PC tissue by immunohistochemistry. Tumors from patients with PC-induced cachexia at diagnosis (n = 13) showed markedly higher expression of S100A8 (P = 0.003) and S100A9 (P < 0.001) compared with tumors of PC patients without cachexia (n = 12; Fig. 3a, b), while S100A8/A9 heterodimer showed no prominent presence in both groups of PC patients and was not further evaluated. In those patients, the correlation between the serum and tumor tissue levels was marginal for S100A8 (r = 0.38, P = 0.060; Fig. 3c), while a significant positive correlation was noted between the serum and tumor tissue levels of S100A9 (r = 0.41, P = 0.042; Fig. 3d). Furthermore, compared with PC patients without cachexia (n = 12), those with cachexia (n = 13) had significantly higher serum levels of S100A8 [median (IQR) 10.4 (7.8–19.8) vs. 6.5 (2.8–11.9) ng/ml, P = 0.022] and numerically higher serum levels of S100A9 [median (IQR): 11.3 (3.0-17.8) vs. 4.0 (1.6–11.5) ng/ml, P = 0.092].

### Associations between blood levels of S100A8, S100A9, S100A8/A9 and cachexia-related parameters

**Fig. 4:**
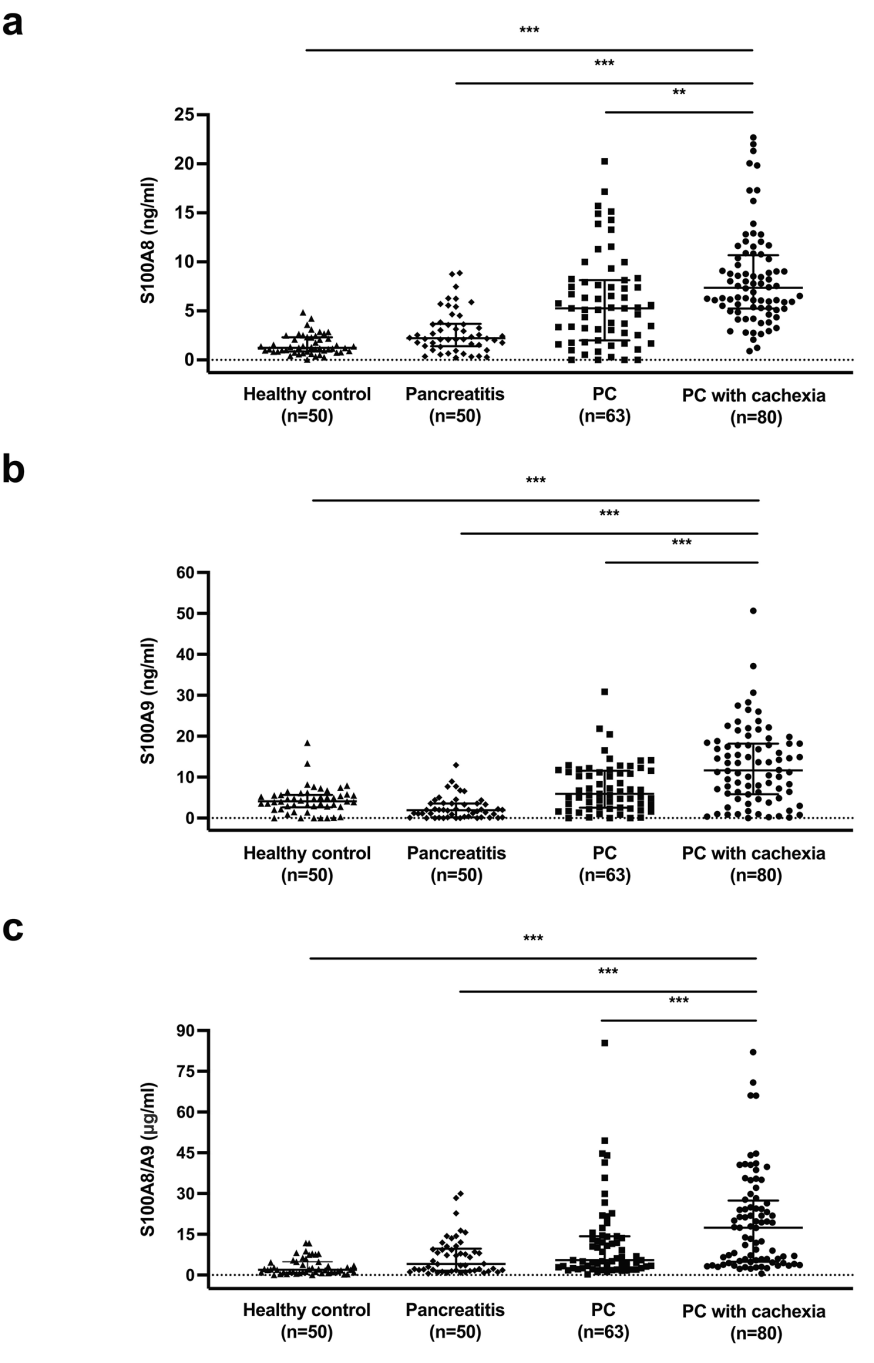
Serum levels of potential cachexigenic factors in healthy, pancreatitis and pancreatic cancer patients with or without cachexia. (a-c) Serum concentrations of S100A8 **(a)**, S100A9 **(b)** and S100A8/A9 heterodimer **(c)** were determined by ELISA. Data are presented as median with interquartile range. PC, pancreatic cancer. **P < 0.01; ***P < 0.001

**Fig. 5:**
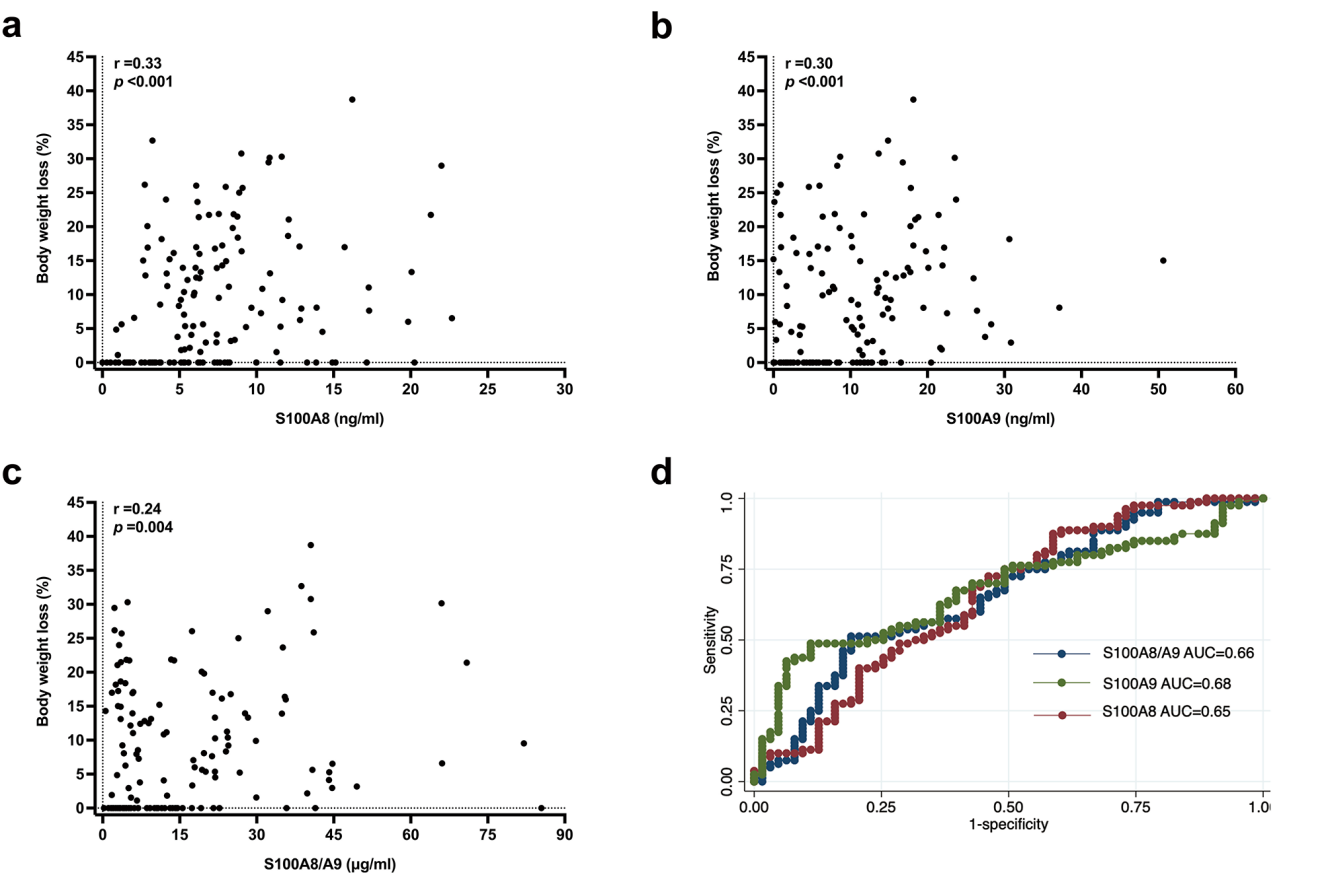
Correlations between serum levels of potential cachexigenic factors and body weight loss and evaluation of differentiation utility in pancreatic cancer patients. **(a-c)** Percentage of body weight loss in pancreatic cancer patients with (n = 80) or without (n = 63) cachexia was correlated with serum concentrations of S100A8 **(a)**, S100A9 **(b)** or S100A8/A9 heterodimer **(c)**, respectively. Spearman correlation coefficient (r) and significance of relevance were evaluated. **(d)** Receiver operating characteristic curves of potential cachexigenic factors for differentiating between pancreatic cancer patients with and without cachexia. Serum levels of S100A8, S100A9 or S100A8/A9 heterodimer in pancreatic cancer patients with (n = 80) and without (n = 63) cachexia were used for evaluation. The area under curves for S100A8, S100A9 and S100A8/A9 were determined

Among 143 patients with PC, 80 (55.9%) had cachexia at cancer diagnosis. The prevalence of cachexia was 50% in stage I, 59.4% in stage II, 57.5% in stage III, and 54.4% in stage IV, without significant differences across stages (P = 0.928; Table 2). Lumbar SMI was significantly lower in cachectic male patients compared with non-cachectic male patients (P = 0.021), whereas no significant differences in SMI were noted between female patients with and without cachexia (P = 0.684). Patients with PC-induced cachexia at diagnosis had significantly higher serum levels of S100A8, S100A9 and S100A8/A9 compared with PC patients without cachexia, patients with pancreatitis, and healthy controls (Table 3; Fig. 4a-c). Furthermore, the percentage of weight loss within 6 months before cancer diagnosis in PC patients positively correlated with serum levels of S100A8 (r = 0.33, P < 0.001), S100A9 (r = 0.30, P < 0.001), and S100A8/A9 (r = 0.24, P = 0.004; Fig. 5a-c). In contrast, the serum level of IL-6 was higher in PC patients with cachexia compared with those without cachexia (P = 0.011) but did not correlate with the percentage of weight loss (r = 0.15, P = 0.069; Supplementary Fig. 1a, c). Serum TNF-α level was not significantly different between PC patients with and without cachexia (P = 0.438) and did not correlate with the percentage of weight loss (r = 0.09, P = 0.294; Supplementary Fig. 1b, d). In addition, serum levels of either S100A8, S100A9, or S100A8/A9 did not correlate with those of IL-6 or TNF-α in PC patients with cachexia (n = 80; Supplementary Fig. 2a-c).

**Table 2:** Characteristics of patients with and without cachexia at diagnosis of pancreatic cancer

	Without cachexia (n = 63)	With cachexia (n = 80)	P value
Age (year), mean ± SD	63.0 ± 12.5	64.8 ± 11.2	0.378
Male, n (%)	40 (63.5)	46 (57.5)	0.496
Stage I/II/III/IV (%)	11.1/20.6/27.0/41.3	8.7/23.7/28.8/38.8	0.928
Primary tumor size (cm)^*^	3.4 (2.7-5.0)	3.7 (3.0–5.0)	0.204
PCDM, n (%)	26 (41.3)	44 (55.0)	0.130
Fasting blood glucose (mg/dL)^*^	113 (96–145)	133 (105–176)	0.051
Body mass index (kg/m^2^), mean ± SD	22.9 ± 3.4	23.0 ± 3.4	0.892
Weight loss (%)^*^	0 (0–0)	12.2 (7.8–17.7)	< 0.001
Lumbar SMI (cm^2^/m^2^)^*^			
Male	46.1 (43.2–51.3)	42.5 (37.6–49.9)	0.021
Female	36.9 (33.5–41.5)	35.6 (31.5–40.7)	0.684
Tumor necrosis factor-α (pg/ml)^*^	16.76 (4.78–48.97)	22.41 (13.32–36.97)	0.438
Interleukin-6 (pg/ml)^*^	5.93 (0-66.13)	41.21 (0-241.83)	0.011

PCDM, pancreatic cancer-associated diabetes mellitus; SMI, skeletal muscle mass index

*Median (interquartile range)

**Table 3:** Serum levels of potential cachexigenic factors in pancreatic cancer patients, pancreatitis and healthy controls

	Control (n = 50)	Pancreatitis (n = 50)		Pancreatic cancer	
Factor, median (interquartile range)				Cachexia (-) (n = 63)	Cachexia (+) (n = 80)
Serum S100A8 (ng/ml)	1.23 (0.86–2.30)		2.22 (1.42–3.63)	5.26 (2.00-8.13)	7.36 (5.26–10.58)^a^
Serum S100A9 (ng/ml)	4.08 (2.76–5.63)		1.92 (0.19–3.56)	5.90 (2.55–11.58)	11.63 (5.89–18.17)^b^
Serum S100A8/A9 (µg/ml)	1.95 (1.05–4.86)		4.06 (1.64–9.45)	5.51 (2.56–14.29)	17.41 (5.00-27.07)^c^

^a^P<0.001 compared with controls, pancreatitis, and PC without cachexia

^b^P<0.001 compared with controls and pancreatitis; P = 0.001 compared with PC without cachexia

^c^P<0.001 compared with controls and pancreatitis; P = 0.001 compared with PC without cachexia

### Predictors of cachexia

Multivariable logistic regression analysis demonstrated that serum levels of S100A8, S100A9, and S100A8/A9 were independently associated with the risk of cachexia [adjusted odds ratio (aOR) 1.11 (95% confidence interval (CI), 1.02–1.21; P = 0.014) per 1 ng/mL increase in serum S100A8, 1.10 (95% CI, 1.04–1.16; P = 0.001) per 1 ng/mL increase in serum S100A9, 1.04 (95% CI, 1.01–1.06; P = 0.009) per 1 µg/mL increase in serum S100A8/A9] (Table 4). In comparison, tumor size, stage, pancreatic cancer-associated diabetes mellitus or fasting blood glucose, and serum levels of TNF-α and IL-6 were not associated with the risk of cachexia. In addition, the area under the curves of the receiver operating characteristic curves for serum levels of the 3 potential cachexigenic factors in differentiating between PC patients with and without cachexia were S100A8: 0.65 (95% CI: 0.56–0.75); S100A9: 0.68 (95% CI: 0.59–0.77), and S100A8/A9: 0.66 (95% CI: 0.57–0.75), respectively (Fig. 5d).

**Table 4:** Predictors of cachexia

	Univariable analysis		Multivariable analysis	
	Odds ratio (95% CI)	P value	Odds ratio (95% CI)	P value
Male	0.78 (0.39–1.53)	0.468	─	─
Age > 60 years	1.33 (0.68–2.61)	0.402	─	─
Stage I (as reference)	1		─	─
Stage II	1.46 (0.41–5.17)	0.556	─	─
Stage III	1.35 (0.40–4.59)	0.627	─	─
Stage IV	1.19 (0.37–3.84)	0.768	─	─
Primary tumor size (per 1 cm increase)	1.19 (0.97–1.46)	0.102	1.13 (0.89–1.42)	0.306
Pancreatic cancer-associated diabetes mellitus	1.74 (0.89–3.39)	0.104	1.23 (0.58–2.61)	0.593
Fasting blood glucose (per 10 mg/dL increase)	1.03 (0.97–1.09)	0.314	─	─
Serum S100A8 (per 1 ng/mL increase)	1.11 (1.03–1.20)	0.005	1.11 (1.02–1.21)	0.014
Serum S100A9 (per 1 ng/mL increase)	1.10 (1.04–1.16)	< 0.001	1.10 (1.04–1.16)	0.001
Serum S100A8/A9 (per 1 µg/mL increase)	1.03 (1.00-1.06)	0.013	1.04 (1.01–1.06)	0.009
Serum tumor necrosis factor-α (per 1 pg/ml increase)	1.00 (1.00–1.00)	0.337	─	─
Serum interleukin-6 (per 1 pg/ml increase)	1.00 (1.00–1.00)	0.770	─	─

## Discussion

This study discovered novel PC-derived cachexigenic factors S100A8, S100A9, and S100A8/A9 heterodimer that potentially mediate muscle wasting, the hallmark of cancer cachexia, and thus have important prognostic and therapeutic implications. Our findings provide further support for the notion that cancer cachexia is a paraneoplastic syndrome triggered by cancer-induced systemic inflammation [14]. S100A8 and S100A9, which usually exist in the form of heterodimers, are members of the calcium binding S100 protein family and play important roles in augmenting inflammation by recruiting leukocytes and inducing cytokine secretion [23]. They also play crucial roles in amplifying the inflammatory response related to infection, autoimmunity, and cancer [26, 27]. In line with our results, previous research has demonstrated significant overexpression (> 10-fold) of S100A8 in pancreatic adenocarcinoma, whereas S100A8 and S100A9 were rarely expressed in normal pancreatic tissue and tissue of chronic pancreatitis [28–30]. Although it is well known that systemic inflammation drives cancer cachexia and S100A8, S100A9, and S100A8/A9 have potent proinflammatory functions, the link between these factors and PC-induced cachexia has not been discovered previously.

The potential causal roles of these factors in PC-induced cachexia were further demonstrated in this study. We showed that these factors could promote skeletal muscle atrophy in vitro and provided new insights into the link between systemic inflammation and sarcopenia. Consistent with previous studies [3], our finding also revealed that the prevalence of cachexia exceeded 50% regardless of tumor stage at the diagnosis of PC, further supporting the notion that PC-induced cachexia is mainly mediated by PC-derived cachexigenic factors. Notably, serum levels of TNF-α and IL-6, the pro-inflammatory cytokines most commonly implicated in cancer cachexia [31] did not correlate with the degree of weight loss or predict the occurrence of cachexia at the diagnosis of PC in this study. Although previous studies showed that patients with cancer cachexia had increased levels of IL-6 and TNF-α compared with control subjects [32, 33], this and other studies [10, 11, 13] have found no significant difference in the levels of IL-6 and TNF-α between PC patients with and without cachexia. Level of monocyte chemoattractant protein-1 has been shown to be marginally increased in cachectic PC patients compared with weight-stable PC patients, but it did not correlate with the degree of weight loss [13]. Taken together, changes in IL-6, TNF-α or other pro-inflammatory factors reported previously might be the consequences of the complex tumor-incited immunological responses mediating cachexia, whereas our results support that S100A8, S100A9 and S100A8/A9 are potential drivers of PC-induced cachexia.

The dual actions of S100A9 in mediating cachexia and diabetes underscore its important roles in PC and potential implications in detection and therapy. Pancreatic cancer-associated new-onset diabetes mellitus (PCDM) and PC-induced cachexia are distinct PC-induced aberration of host metabolism to supply nutrients such as amino acids and glucose for tumor growth [8]. We previously discovered that PC-derived S100A9 could induce insulin resistance by inhibiting insulin-stimulated glucose uptake in muscle cells and thereby led to PCDM [16], a paraneoplastic phenomenon occurring in approximately 50% of PC patients within 24 months before the diagnosis of cancer [34]. Whereas in a previous study the association between serum S100A9 level and cachexia-related parameters was not statistically significant [35], this study included more PC patients (n = 143) compared with the previous study (n = 88), resulting in greater statistical power. The combined in-vitro and clinical findings provide strong evidence that S100A9 contributes to PC-induced cachexia. PCDM has been recognized as a window of opportunity for early detection as the onset of PCDM is generally at the early stage of PC [34], and we showed that blood levels of PC-derived diabetogenic factors could distinguish PCDM from type 2 diabetes among patients with new-onset diabetes.[16] Similarly, this and previous studies [3] consistently report a high prevalence of cachexia in early stage of PC (50% in stage I in this study), and wasting of body protein with elevated circulating amino acids preceded cancer diagnosis by 2 to 5 years in PC patients [9]. Therefore, blood levels of the identified cachexigenic factors might help identify early PC individuals with unexplained weight loss, and therapies targeting S100A9 activity might attenuate both PC-induced cachexia and PCDM, reducing PC-induced complications.

Besides collecting information on weight loss, we also accurately measured skeletal muscle mass in the study, enabling accurate determination of the cachexia status in PC patients. We focused on cachexia at the time of diagnosis which was mainly attributed to PC-derived cachexigenic factors, reducing confounding by factors that may induce or exacerbate cachexia later in the disease course, such as anti-cancer treatment and gastrointestinal obstruction. However, information on skeletal muscle mass before the onset of PC was not available; therefore, we could not examine whether blood levels of the identified cachexigenic factors were also correlated with the degree of muscle mass loss. Given that measurement of skeletal muscle mass is seldom performed for healthy individuals, information on usual muscle mass is unlikely to be available. However, we did note that blood levels of the identified cachexigenic factors positively correlated with the degree of weight loss for which muscle wasting accounts a major portion in cancer cachexia [4, 9], suggesting potential positive correlations with the degree of muscle mass loss. Second, the relative importance of the 3 identified cachexigenic factors in mediating PC-induced cachexia remained to be determined. Given that S100A8/A9 is the predominant form in the circulation and the serum level of S100A8/A9 observed in this study was three orders of magnitude higher than those of S100A8 and S100A9, S100A8/A9 heterodimer may be the most promising candidates among the three cachexigenic factors.

## Conclusion

In conclusion, in vitro atrophic effects of S100A8, S100A9, and S100A8/A9 suggest their potential as pathogenic factors of PC-induced cachexia. Moreover, the positive correlation with the degree of weight loss and their ability to predict cachexia in PC patients using clinical specimens imply their potential as diagnostic markers for PC-induced cachexia. Further exploration of the significance and application of S100A8, S100A9 and S100A8/A9, as well as elucidation of their mechanistic roles, could make a promising contribution to the advances of PC detection and clinical management.

## Electronic supplementary material

Below is the link to the electronic supplementary material.

Supplementary Material 1

Supplementary Material 2

## Data Availability

TCGA pancreatic cancer and GTEx normal pancreas RNAseq datasets analysis in this study is From GEPIA (http://gepia.cancer-pku.cn). Secretome list is retrieved from Human Protein Atlas (https://www.proteinatlas.org/humanproteome/tissue/secretome). Blots raw images are provided in Supplementary Figs. 3–7. Other data used and/or analyzed during the current study would be available from the corresponding author on reasonable request.

## Abbreviations

Atrogin-1: Muscle atrophy F-box protein
C2C12: Mouse myoblast cell line
CI: Confidence interval
GTEx: Genotype-tissue expression
h: hour
H-score: Histochemical scoring
IL-6: Interleukin-6
IQR: Inter-quartile range
MIA PaCa-2: Human pancreatic ductal adenocarcinoma cell line
MuRF1: Muscle-specific RING finger protein 1
OR: Odd ratio
PC: Pancreatic cancer
PCDM: Pancreatic cancer-associated new-onset diabetes mellitus
S100A8: S100 calcium-binding protein A8
S100A9: S100 calcium-binding protein A9
SD: Standard deviation
TNFα: Tumor necrosis factorα
TCGA: The cancer genome atlas
