# Supplementary material for: S100A8, S100A9 and S100A8/A9 heterodimer as novel cachexigenic factors for pancreatic cancer-induced cachexia
Source: BMC Cancer. 2023 Jun 6;23:513. doi: 10.1186/s12885-023-11009-8 (PMC10242984; doi:10.1186/s12885-023-11009-8)

**Online Supplemental Information**

**S100A8, S100A9 and S100A8/A9 Heterodimer as Novel Cachexigenic Factors for Pancreatic Cancer-Induced Cachexia**

Wei-Chih Liao, MD, PhD^1,2^, Chih-Ta Chen PhD^3^, You-Shu Tsai MS^3^, Xin-Ya Wang MS^3^, Yen-Tzu Chang MS^3^, Ming-Shiang Wu, MD, PhD^1,2^, Lu-Ping Chow, PhD^3*^

^1^Division of Gastroenterology and Hepatology, Department of Internal Medicine, National Taiwan University Hospital, Taipei, Taiwan

^2^Department of Internal Medicine, College of Medicine, National Taiwan University, Taipei, Taiwan

^3^Graduate Institute of Biochemistry and Molecular Biology, College of Medicine, National Taiwan University, Taipei, Taiwan

***Correspondence:**

Lu-Ping Chow, Graduate Institute of Biochemistry and Molecular Biology, College of Medicine, National Taiwan University, No.1, Jen-Ai Road Section 1, Taipei 10051 Taiwan. Email: [chowip@ntu.edu.tw](mailto:chowip@ntu.edu.tw). Fax: (886)2-23958814. Telephone: (886)2-23123456-88214.

**Supplementary tables**

| **Supplementary Table S1. Biological process enrichment of upregulated pancreatic cancer-secreted molecules (n=416) via Database for Annotation, Visualization and Integrated Discovery** | | | |
| --- | --- | --- | --- |
| **Biological process** | **No. of genes** | **P value** | **Genes** |
| Inflammatory response | 50 | 6.7E-23 | CCL13, CCL15, CCL17, CCL18, CCL19, CCL20, CCL21, CCL22, CCL24, CCL26, CCL3, CCL4L2, CCL4, CXCL1, CXCL10, CXCL13, CXCL3, CXCL5, CXCL6, CXCL8, CXCL9, CRP, CD14, S100A8, S100A9, TNFAIP6, TNFRSF11B, TNFRSF6B, AOAH, AOC3, ANXA1, BMP2, CHI3L1, C3, C4B, ECM1, LGALS9, HYAL1, IGFBP4, IL15, IL18, IL34, LY86, LY96, LYZ, MIF, SPP1, SCG2, THBS1, TGFB1. |
| Chemokine-mediated signaling pathway | 22 | 5.4E-18 | CCL13, CCL15, CCL17, CCL18, CCL19, CCL20, CCL21, CCL22, CCL24, CCL26, CCL3, CCL4L2, CCL4, CXCL1, CXCL10, CXCL13, CXCL3, CXCL5, CXCL6, CXCL6, CXCL9, TFF2. |
| Cellular response to tumor necrosis factor | 25 | 5.0E-17 | ADAMTS12, ADAMTS7, CCL13, CCL15, CCL17, CCL18, CCL19, CCL20, CCL21, CCL22, CCL24, CCL26, CCL3, CCL4L2, CCL4, CXCL8, CHI3L1, COL1A1, EDN1, HYAL1, IL18BP, LCN2, NPNT, POSTN, THBS1. |
| Cellular response to interleukin-1 | 21 | 9.7E-17 | ADAMTS12, CCL13, CCL15, CCL17, CCL18, CCL19, CCL20, CCL21, CCL22, CCL24, CCL26, CCL3, CCL4L2, CCL4, CXCL8, CHI3L1, EDN1, HYAL1, LCN2, TFPI. |
| Immune response | 44 | 2.4E-16 | CCL13, CCL15, CCL18, CCL19, CCL20, CCL21, CCL22, CCL24, CCL26, CCL3, CCL4L2, CCL4, CXCL1, CXCL10, CXCL13, CXCL14, CXCL3, CXCL5, CXCL6, CXCL8, CXCL9, TNFRSF11B, TNFRSF6B, CTSW, CHIT1, C1QC, C1R, C3, CST7, GZMA, GZMH, IL1RN, IL15, IL16, IL18, JCHAIN, LIF, OSM, PXDN, SLPI, SEMA3C, THBS1, TINAGL1. |
| Positive regulation of ERK1 and ERK2 cascade | 28 | 5.8E-15 | CCL13, CCL15, CCL17, CCL18, CCL29, CCL20, CCL21, CCL22, CCL24, CCL26, CCL3, CL4L2, CCL4, BMP2, CHI3L1, CTGF, FGA, FGG, FGF1, FGF19, LGAF19, LGALS9, MIF, NPNT, PDGFC, PDGFB, TGFB1, VEGFB. |

**Supplementary figure legends**

**Supplementary Figure 1.** Serum levels of IL-6 and TNFα and correlations with body weight loss in pancreatic cancer (PC) patients with or without cachexia. (a, b) Serum concentrations of IL-6 (a) and TNFα (b) were determined by ELISA. Data are presented as median with interquartile range. (c, d) Percentage of body weight loss in pancreatic cancer patients with (n=80) or without (n=63) cachexia was correlated with serum concentrations of IL-6 (c) and TNFα (d), respectively. Spearman correlation coefficient (r) and significance of relevance were evaluated. ns, not significant; *P<0.05.

**Supplementary Figure 2.** Correlations between serum levels of potential cachexigenic factors and IL-6 or TNFα in patients with pancreatic cancer-induced cachexia. (a-c) Serum concentrations of S100A8 (a), S100A9 (b) or S100A8/A9 heterodimer (c) were correlated with that of IL-6 or TNFα respectively in cachectic pancreatic patients (n=80). Spearman correlation coefficient (r) and significance of relevance were evaluated.


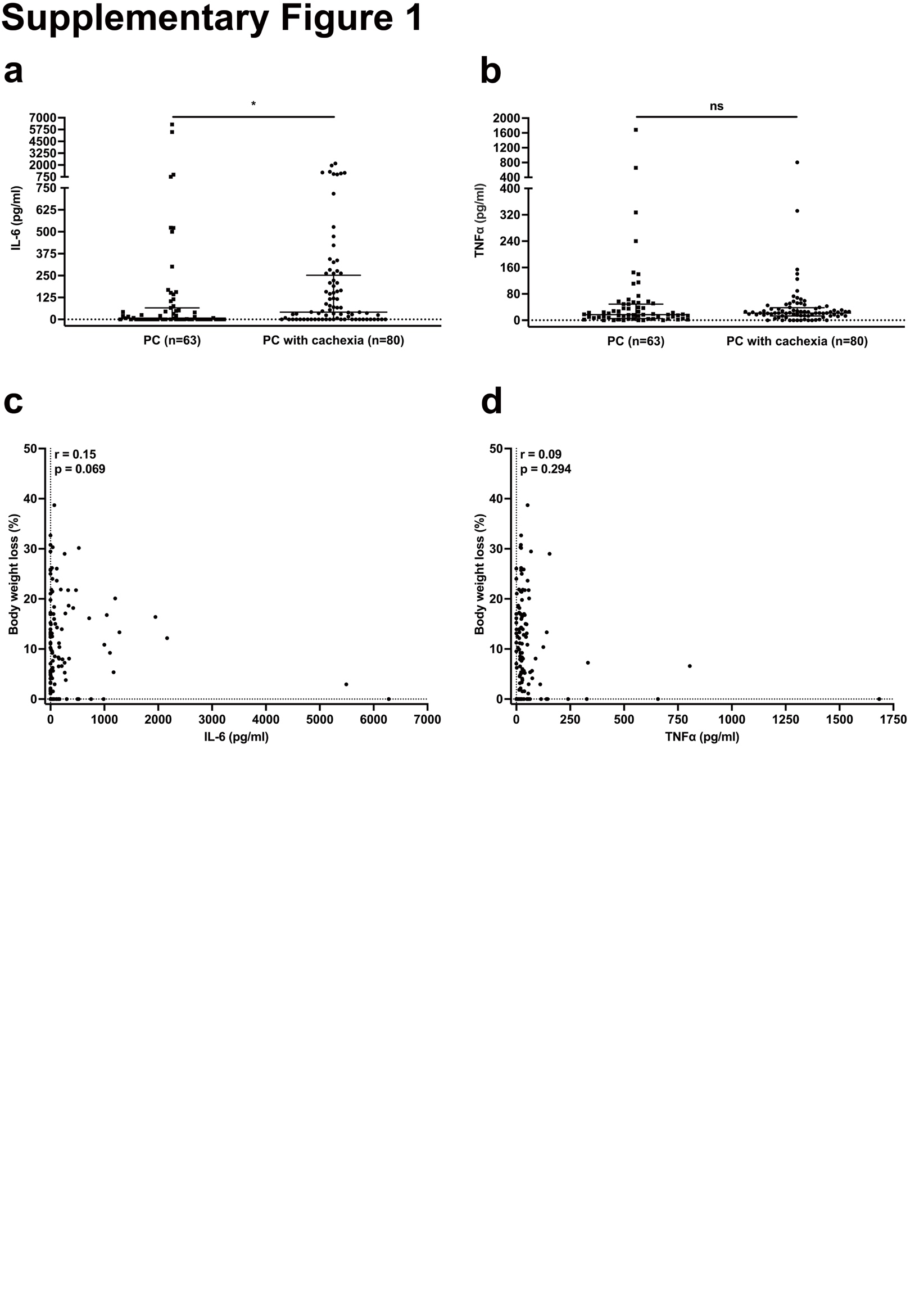


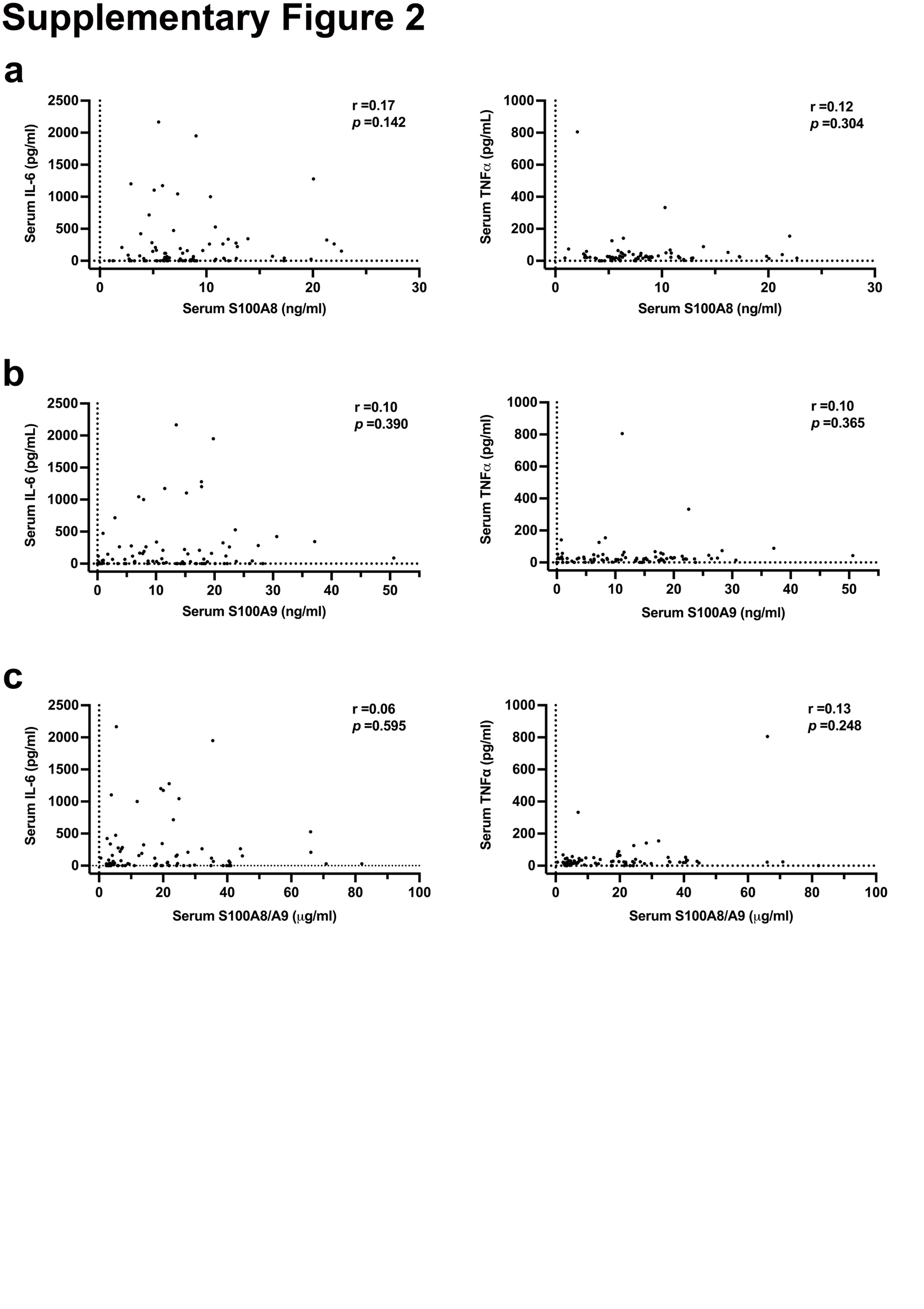

Supplement: Supplementary file 1 — Supplementary Material 1 [file 12885_2023_11009_MOESM1_ESM.docx]
