# Supplementary material for: S100A8, S100A9 and S100A8/A9 heterodimer as novel cachexigenic factors for pancreatic cancer-induced cachexia
Source: BMC Cancer. 2023 Jun 6;23:513. doi: 10.1186/s12885-023-11009-8 (PMC10242984; doi:10.1186/s12885-023-11009-8)
Supplement: Supplementary file 2 — Supplementary Material 2 [file 12885_2023_11009_MOESM2_ESM.pdf]

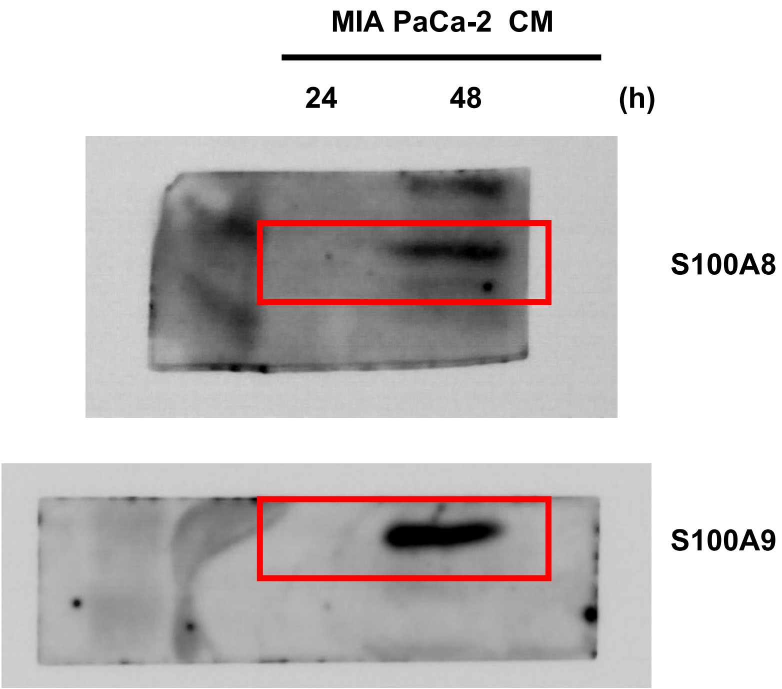

Supplementary Fig. 3 Blots raw image for Fig.1e. The corresponding bands are indicated by red boxes.

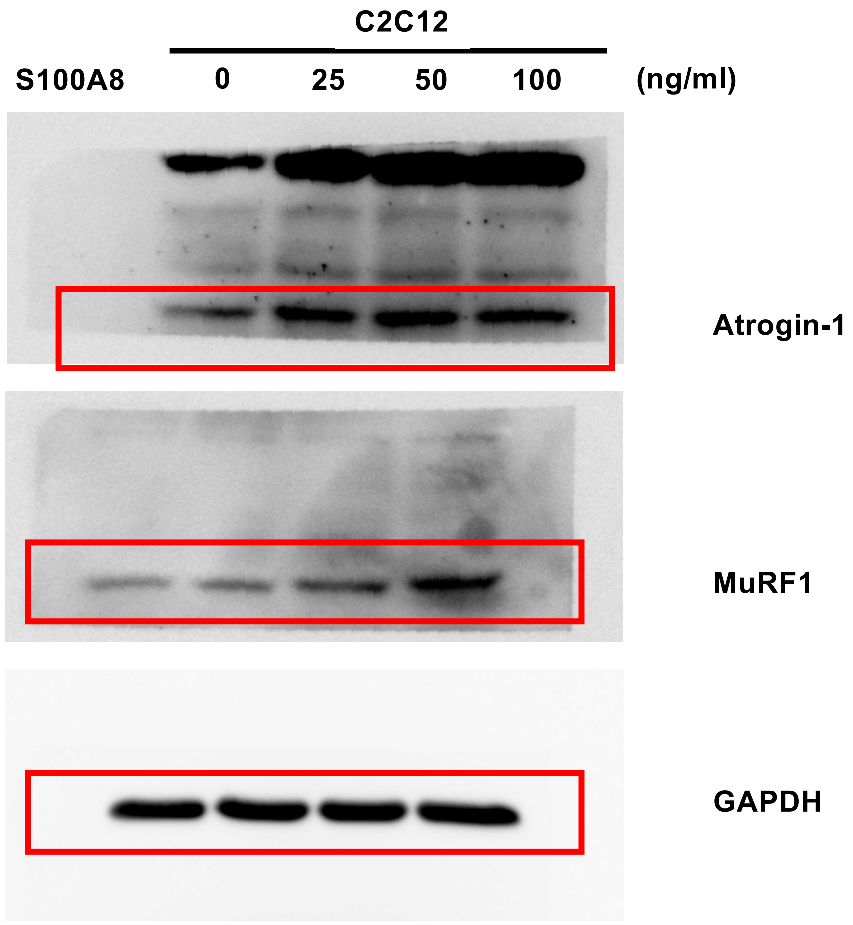

Supplementary Fig. 4 Blots raw image for Fig. 2a. The corresponding bands are indicated by red boxes.

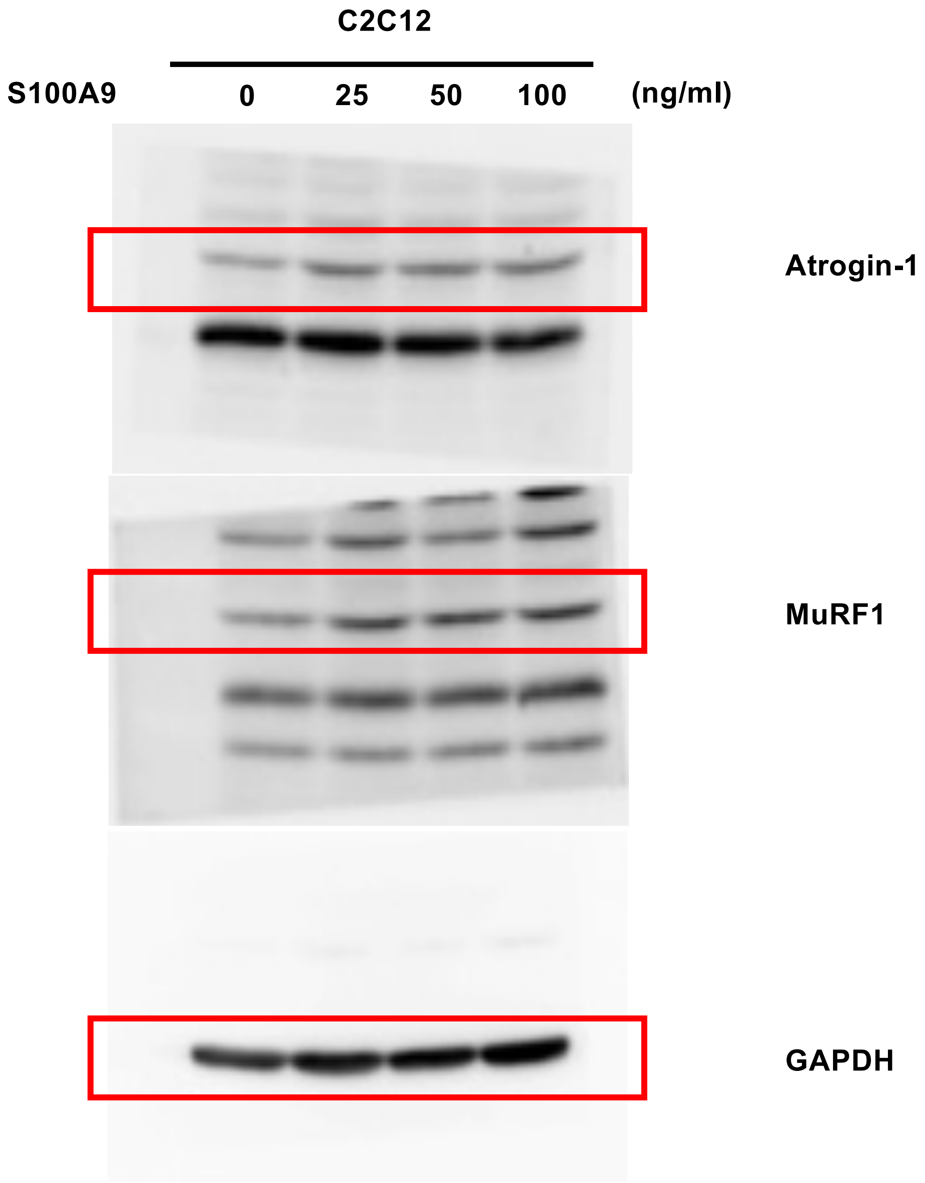

Supplementary Fig. 5 Blots raw image for Fig. 2b. The corresponding bands are indicated by red boxes.

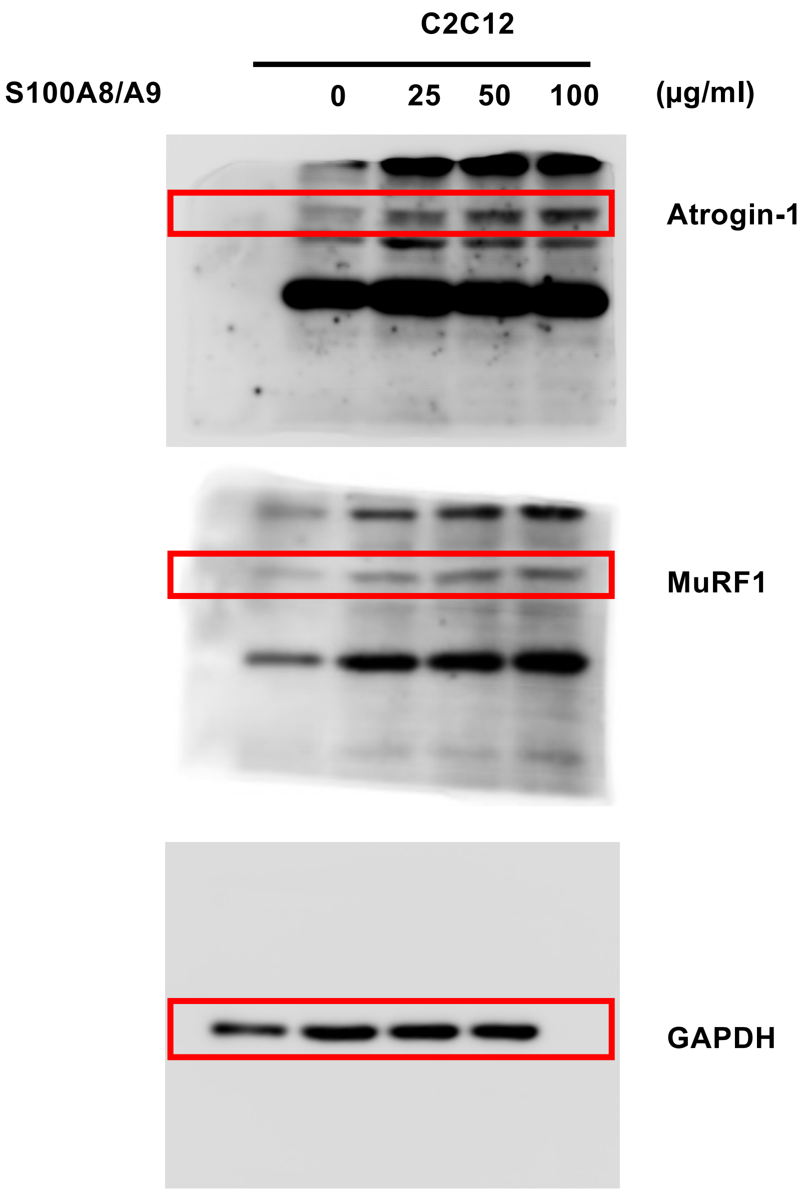

Supplementary Fig. 6 Blots raw image for Fig. 2c. The corresponding bands are indicated by red boxes.

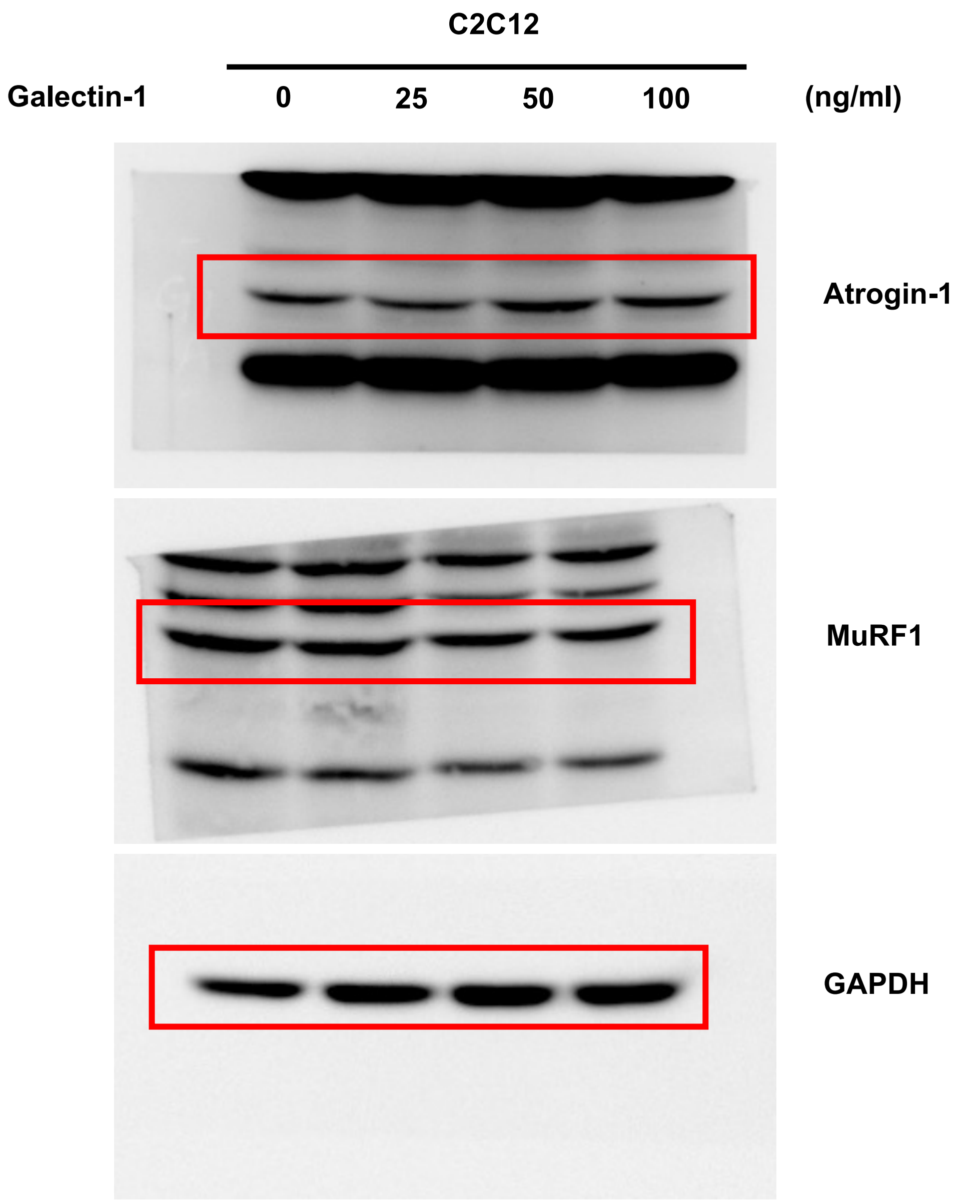

Supplementary Fig. 7 Blots raw image for Fig. 2d. The corresponding bands are indicated by red boxes.
